# Dilute but Dense – Reversible Crosslinking Enables Water‐Rich (Bio)polymer Condensates

**DOI:** 10.1002/advs.202519636

**Published:** 2026-02-04

**Authors:** Xinxiang Chen, Jude Ann Vishnu, Pol Besenius, Julian König, Friederike Schmid

**Affiliations:** ^1^ Institute of Physics Johannes Gutenberg‐University Mainz Mainz Germany; ^2^ LPTMS, Université Paris‐Saclay France; ^3^ Department of Chemistry Johannes Gutenberg‐University Mainz Germany; ^4^ Theodor Boveri Institute, Biocenter University of Würzburg Würzburg Germany

**Keywords:** liquid–liquid phase separation, RNA‐protein solution, Semenov–Rubinstein theory, sol‐gel transition

## Abstract

Liquid–liquid phase separation (LLPS) of polymers underlies the formation of biomolecular condensates and offers a versatile route to functional soft materials. Traditionally, LLPS is attributed to changes in solvent quality or associative coacervation, but here a purely entropic connectivity‐driven mechanism is demonstrated: reversible crosslinking. Using coarse‐grained simulations of a minimal bead–spring model in good solvent, it is shown that transient, pairwise crosslinks alone can drive phase separation at ultralow polymer densities, yielding highly swollen, water‐rich condensates. The phase behavior exhibits closed‐loop coexistence and re‐entrant percolation. This is captured quantitatively by a mean‐field Semenov–Rubinstein theory with a single fit parameter, the effective repulsion parameter. Notably, phase boundaries are largely robust to rearrangements of crosslinkable domains along the sequence; only highly blocky sequences appreciably reduce the phase separation region and can even convert condensates into micelles or connected micelle networks. These results establish an entropy‐enabled mechanism for mesoscale organization and suggest routes to programmable, membraneless materials in synthetic and RNA‐protein contexts.

## Graphical abstract

Reversible crosslinking between two types of (bio)polymers drives liquid–liquid phase separation even in good solvent. The arrangement of binding motifs controls condensate formation and density, and internal network structure. Simulations and theory reveal a closed‐loop coexistence phase diagram at very low monomer concentrations and re‐entrant gelation.

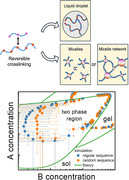

## 1 Introduction

Polymer–solvent phase separation is a ubiquitous phenomenon that shapes materials, biological systems, and industrial processes alike. Its ability to transform molecular‐scale interactions into large scale organization makes it a central principle of soft matter and a powerful route to functional structure. In biological systems, this process manifests as liquid–liquid phase separation (LLPS), which drives the formation of membraneless organelles such as stress granules, nucleoli, and processing bodies [1, 2, 3, 4, 5, 6, 7]. These condensates, typically composed of RNA and proteins, regulate essential cellular functions [8, 9], and can adopt diverse physical states ranging from liquid‐like droplets to more gel‐like assemblies [10, 11]. Their formation and properties depends on diverse factors such as environmental conditions [12, 13], chain composition [14] and charge patterning [15, 16, 17, 18], and deciphering these rules is key not only for cell biology but also for guiding the design of synthetic systems that exploit phase separation for function.

From a polymer science perspective, phase separation is classically understood to arise from two main mechanisms. One is change in solvent quality, where shifts in temperature, composition, or cosolvent conditions alter polymer–solvent interactions and trigger demixing [19, 20]. The other is coacervation, in which associative interactions between polymers – commonly electrostatics or hydrogen bonding – drive the formation of dense polymer‐rich phases [21, 22, 23, 24, 25]. These mechanisms have provided a framework for interpreting both synthetic and biological condensates.

Reversible crosslinking provides a distinct and largely unexplored mechanism for phase separation [26, 27]. Here, reversible crosslinking refers to the formation of very strong but transient bonds with sufficiently high binding energies to allow maximal binding between crosslinkable units, limited only by stoichiometry. In this scenario, the many possible ways how crosslinks between polymer chains can reorganize generate combinatorial entropy that can nucleate and stabilize condensates. Condensation can occur even in good solvent, enabling the assembly of ultra‐dilute, highly hydrated condensates that are qualitatively distinct from classical denser coacervates. The existence of such a mechanism was predicted decades ago by Semenov and Rubinstein [28], but direct realizations have been sparse: Prior studies have shown reversible disulfide bonds assisting LLPS in designer peptides, yet still relied on additional “stickers” or associative motifs [29]. On the computational side, Harmon et al. [30] demonstrated that directed bonds can induce phase separation in poor or marginal solvent. More recently, Rovigatti and Sciortino reported crosslink‐driven phase separation in good solvent, which however required multiple crosslink species [31, 32].

On the other hand, recent experimental work has demonstrated that RNA can drive demixing transitions into highly permeable condensates with densities similar to the surrounding nucleoplasm [33]. Such findings highlight that condensates do not need to be dense to be functionally relevant, and motivate the development of molecular models capable of capturing phase behavior in this water‐rich regime.

Here, we present a minimal system in which reversible crosslinking with a single crosslink type drives phase separation in good solvent, without other associative motifs. These condensates form in quantitative agreement with the Semenov–Rubinstein theory and display ultra‐high water contents – one order of magnitude higher than conventional biomolecular condensates, which may already contain up to 70 volume percent of water [34, 35] due to the high hydration level of biopolymers. Importantly, crosslink‐driven phase separation is largely insensitive to sequence details, with the fraction of crosslinking monomers emerging as the sole control parameter. This stands in contrast to solvent‐quality and coacervation routes, where charge patterning and monomer sequence critically influence phase boundaries and condensate morphologies [24, 36, 37, 38, 39, 40, 41, 42, 43, 44, 45].

Taken together, our findings establish reversible crosslinking as a complementary and theoretically long‐predicted phase separation mechanism alongside solvent‐quality changes and coacervation. This mechanism enables phase separation in good solvent and introduces a robust, tunable pathway to mesoscale organization that expands the design space for synthetic materials and offers new perspectives on how cells might regulate highly dilute biomolecular assemblies.

## 2 Results

**FIGURE 1:**
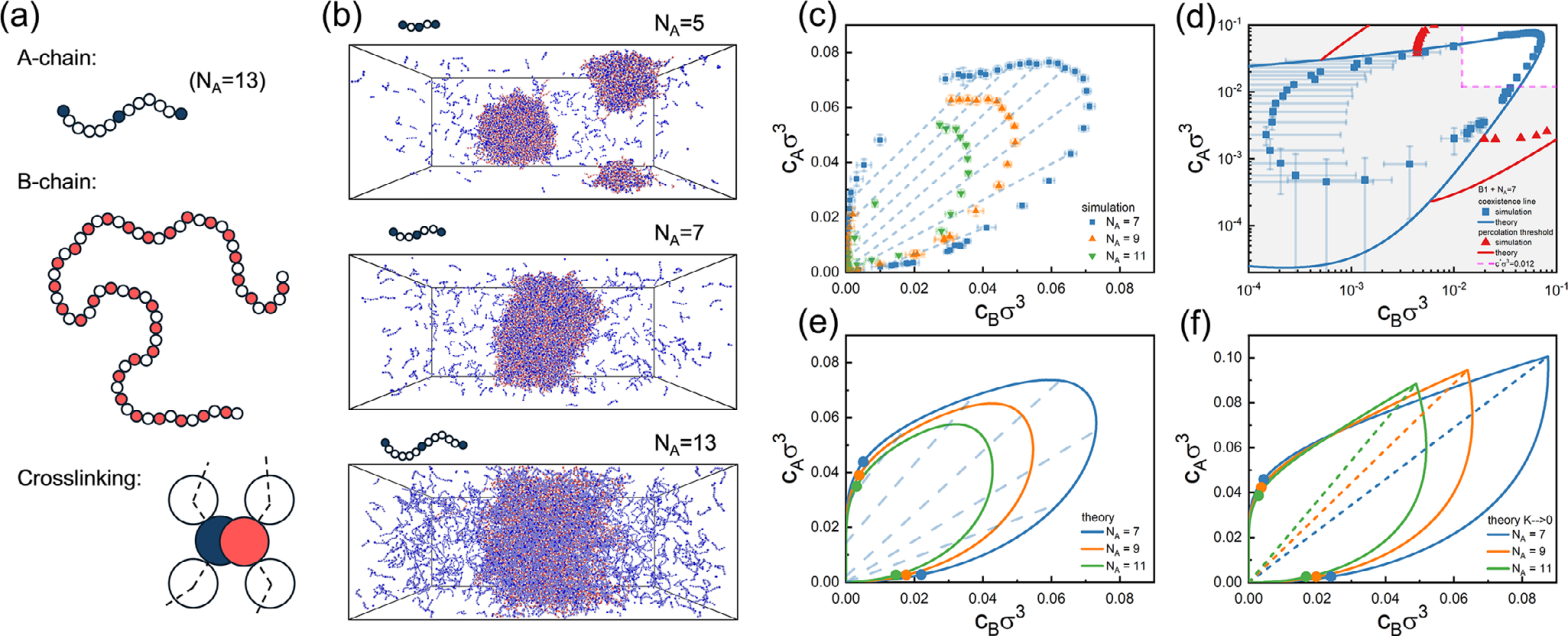
(a) Schematic illustration of the basic model (case $N_{A}=13$): Spring bead chains containing neutral monomers (white) and crosslinkable monomers (red and blue). Crosslinkable monomers can bind exclusively to a single other crosslinkable monomer of opposite type. (b) Snapshots showing phase separation equilibrium in systems of size in a simulation box of size $100\times 200\times 80\sigma^{3}$ (periodic boundary conditions) containing 2140 A‐chains and 200 B‐chains for $N_{A}=5$ (top), $N_{A}=7$ (middle), and $N_{A}=13$ (bottom). (c) Phase diagrams as obtained from simulations for different $N_{A}$ as indicated. Blue dashed lines show tie lines for $N_{A}=7$. (d) Same data (symbols) as (c) for the case $N_{A}=7$ in double logarithmic representation compared to the prediction of the Danielsen–Semenov–Rubinstein theory (lines) with $K=0.001257\sigma^{-3}$ and (fitted) $v_{\text{ex}}=1.73\sigma^{3}$. Red symbols and lines show simulation and theory results for the percolation line. The shaded area indicates the region where the criterion $c_{i}<c^{*}$ is not fulfilled (see text). (e) Theoretical phase diagrams for all $N_{A}$ at $K=0.001257\sigma^{-3}$ and $v_{\text{ex}}=1.73\sigma^{3}$ with tie lines. (f) Corresponding theoretical phase diagram at K→0. The dashed lines indicate stoichiometric conditions, $c_{A}f_{A}/N_{A}=c_{B}f_{B}/N_{B}$, where all crosslinkable monomers are bound and none are in excess.

We considered a mixture of two polymer species A and B of length $N_{A}$ and $N_{B}$ in implicit solvent, each containing $f_{A}$ and $f_{B}$ crosslinkable monomer units, respectively, which can reversibly react with each other to form one‐to‐one bonds (see Figure 1a). Apart from that, all monomers interact with repulsive potentials, corresponding to the effect of a good solvent. Specifically, we used a bead‐spring polymer model with an additional crosslinking potential between crosslinkable monomers [46]. This model is similar in spirit to the popular sticker‐spacer models for intrinsically disordered proteins [47] in a variant where “stickers” can only bind in pairs [48], which is additionally designed to explicitly represent good solvent conditions. Details of the simulation model and method can be found in the Experimental Section. The model parameters used in this work are $f_{A}=3,\;N_{A}\in \left[5,7,9,11,13,23\right],\;f_{B}=32,\;N_{B}=65$. The crosslinkable A‐units were positioned at the two ends and in the middle of A‐chains, and unless stated otherwise, the B‐crosslinkers were distributed evenly on B‐chains as shown in Figure 1a. In the following, we will report energies in units of the thermal energy $k_{B}T$ and lengths in units of the monomer diameter σ. In these units, the crosslinking energy was set to $6k_{B}T$ corresponding to a dissociation constant of $K=0.001257\;\sigma^{-3}$. The choice of the binding strength was constrained by the need to keep equilibration times within accessible simulation time scales. Therefore, it had to be chosen relatively weak, such that crosslinks are not fully saturated (see Figure S4b in Supporting Information). To assess the impact of incomplete saturation, we will also consider the limiting case K→0 at the theoretical level in the discussion below.

### 2.1 Phase Separation and Phase Behavior

For sufficiently short A‐chains, the system described above undergoes phase separation at low monomer concentrations, as illustrated by example simulation snapshots in Figure 1b. As the A‐chain length is increased – corresponding to a higher fraction of neutral monomers, the condensate swells. For longer chains, ($N_{A}=23$), phase separation is entirely suppressed, consistent with previous findings [46] (data not shown). To quantify the phase behavior, we performed slab simulations and determined the densities of the dense and dilute phases from the resulting density profiles, averaging over 4–10 independent simulations (see Experimental Section for details). Representative phase diagrams are shown in Figure 1c. The influence of finite size effects on the results for the phase boundaries is tested in Figure S5 in Supporting Information for $N_{A}=7$ and found to be negligible. Therefore, all phase diagrams reported here and in the following were obtained with the fixed box size $30\times 300\times 30\sigma^{3}$. Notably, in these diagrams, the monomer number density in the coexisting dense phase can be as low as $0.04\sigma^{-3}$, corresponding to highly swollen gels with polymer packing fractions of only 2%.

The system displays closed‐loop phase behavior, similar to that observed in polyelectrolyte complex coacervation under nonstoechiometric conditions [49, 50]. Phase separation is driven by the formation of dense droplets which are stabilized by numerous A‐chain mediated bridging interactions between B‐chains and vice versa. Figure S6 in Supporting Information shows that the number of bridges in the system initially increases with the concentration of the A component and then decreases again. When the A concentration is either too low or too high, bridging becomes too infrequent to support condensation: If A is scarce, too few A‐chains are available to serve as bridges. Conversely, if A is too much in excess, most A‐chains only bind once to a B‐chain due to the limited availability of crosslinking partners. In both cases, bridging interactions are insufficient to sustain phase separation. Consequently, upon increasing the A concentration at fixed B concentration, the system exhibits reentrant behavior, transitioning from a homogeneous phase to phase separation and back to a homogeneous phase.

The behavior of the system is also influenced, to some extent, by chain flexibility. While the total number of crosslinks is largely independent of chain stiffness (see Figure S1 in Supporting Information), stiffness modulates the binding topology: increased stiffness suppresses loop formation and promotes bridging. For highly flexible chains with short spacers, ultrashort loops dominate, and phase separation is replaced by the formation of multiple disconnected A–B complexes (see Supporting Information). In the present work, the chain stiffness parameter is chosen to preclude such ultrashort loop formation.

A second cooperative process is percolation, defined as the emergence of a system‐spanning network. In our slab simulations, we identify percolation by requiring that a continuous path within a connected network links a particle to one of its periodic images [46]. Figure 1d shows the location of the percolation line within the homogeneous region of the phase diagram for the case $N_{A}=7$. Figure S7 in Supporting Information demonstrates that finite‐size effects on the percolation threshold are negligible if the box is cubic. In contrast, simulations performed in a slab geometry with an elongated axis suggest a noticeable shift in the apparent percolation threshold in strongly anisotropic boxes, particularly in the dilute regime, even if the length of the shortest side remains comparable to the side length of the cubic boxes. We attribute this somewhat unintuitive observation to the effect of density fluctuations along the long axis of the box, which can also be seen by visual inspection of the simulation snapshots (see Figure S7c in Supporting Information). Therefore, in Figure 1d, we report the percolation threshold obtained from cubic‐box simulations. The resulting percolation threshold coincides with a sharp decrease in the size of the second‐largest cluster size (see Figure S8 in Supporting Information).

Analogous to phase separation, the system exhibits reentrant percolation behavior, i.e., it may transition from sol (unpercolated) to gel (percolated) and then back to sol upon increasing the concentration of one component [46]. In phase separated configurations, the coexisting dense phase is always a gel as one would expect. Interestingly, the percolated region extends into the dilute phase far enough that close to the low‐concentration critical demixing point, both the coexisting dense and dilute phases can be percolated. A similar gel–gel phase separation phenomenon has recently been observed experimentally during the (irreversible) synthesis of PEG hydrogels from tetra‐functional PEG precursors [51].

Applying a recent extension of the Rubinstein–Semenov theory [52], we also calculated the theoretical prediction for the phase diagram in mean‐field approximation (Figure 1e). The theory balances the translational entropy of A‐ and B‐chains, the excluded volume interactions of monomers, and a crosslink free energy contribution that accounts for both the binding energy and a combinatorial entropy associated with pairing all crosslinkable A‐ and B‐monomers. It does not account for spatial correlations arising from, e.g., chain connectivity and conformations. More details and the relevant equations can be found in Supporting Information.

Despite its simplicity, the theory reproduces the simulation phase boundaries surprisingly well with only one adjustable parameter, the effective repulsion parameter $v_{\text{ex}}$. A naïve estimate based on the second viral coefficient of free monomers gives [46] $v_{\text{ex}}=2.2\sigma^{3}$. Adjusting $v_{\text{ex}}$ to the simulations resulted in a smaller value, $v_{\text{ex}}=1.73\sigma^{3}$, consistent with the expectation that the effective repulsion parameter is reduced for connected monomers. Figure S9 in Supporting Information shows the phase diagrams for different systems, where simulation data are directly compared with theoretical curves in each subfigure. The theory also qualitatively captures the percolation behavior of the system, although the quantitative agreement is less accurate, in line with previous results [46].

Regarding phase boundaries and binding numbers (see Figure S4 in Supporting Information), the theoretical predictions agree remarkably well with simulations, far better than expected. Semenov and Rubinstein argued that their mean‐field approximation should apply when excluded volume interactions are weak on the spacer scale and spacers overlap [28, 52]. Neither condition is met in our systems, where B‐chains have with a spacer length l=1. The range of validity of the theory therefore appears to be substantially broader than originally anticipated by its authors. As an alternative guideline, we propose considering the overlap concentration of “minimal clusters” comprising one A‐ and B‐chain, estimated from simulations as the overlap concentration of the longer B‐chains $c^{*}=c_{B}^{*}=N_{B}/\left(\frac{4}{3}\pi R_{g,B}^{3}\right)∼0.012\sigma^{-3}$. Figure S4 (Supporting Information) and Figure 1d show that the theoretical predictions are accurate for $c_{A},c_{B}>c^{*}$ and deviations deviations emerge in the dilute regime where $c_{A}<c^{*}$ or $c_{B}<c^{*}$.

In the simulations, we employ a moderate binding energy to accelerate the equilibration of the system. To establish phase separation under strong binding and test whether ultra‐low‐density condensates persist, we examine the athermal limit, $\varepsilon_{\mathrm{sp}}\to \infty$ (equivalently K→0), at the theoretical level. The corresponding theory is derived in Supporting Information. As shown in Figure 1f, the phase boundaries expand slightly compared to those obtained for $\varepsilon_{\mathrm{sp}}=6k_{B}T$ (Figure 1e), indicating that the phase separation mechanism is the same and indeed driven by entropy and connectivity. Importantly, the dense phase remains highly dilute also in the athermal limit.

### 2.2 Robustness under Sequence Variations

**FIGURE 2:**
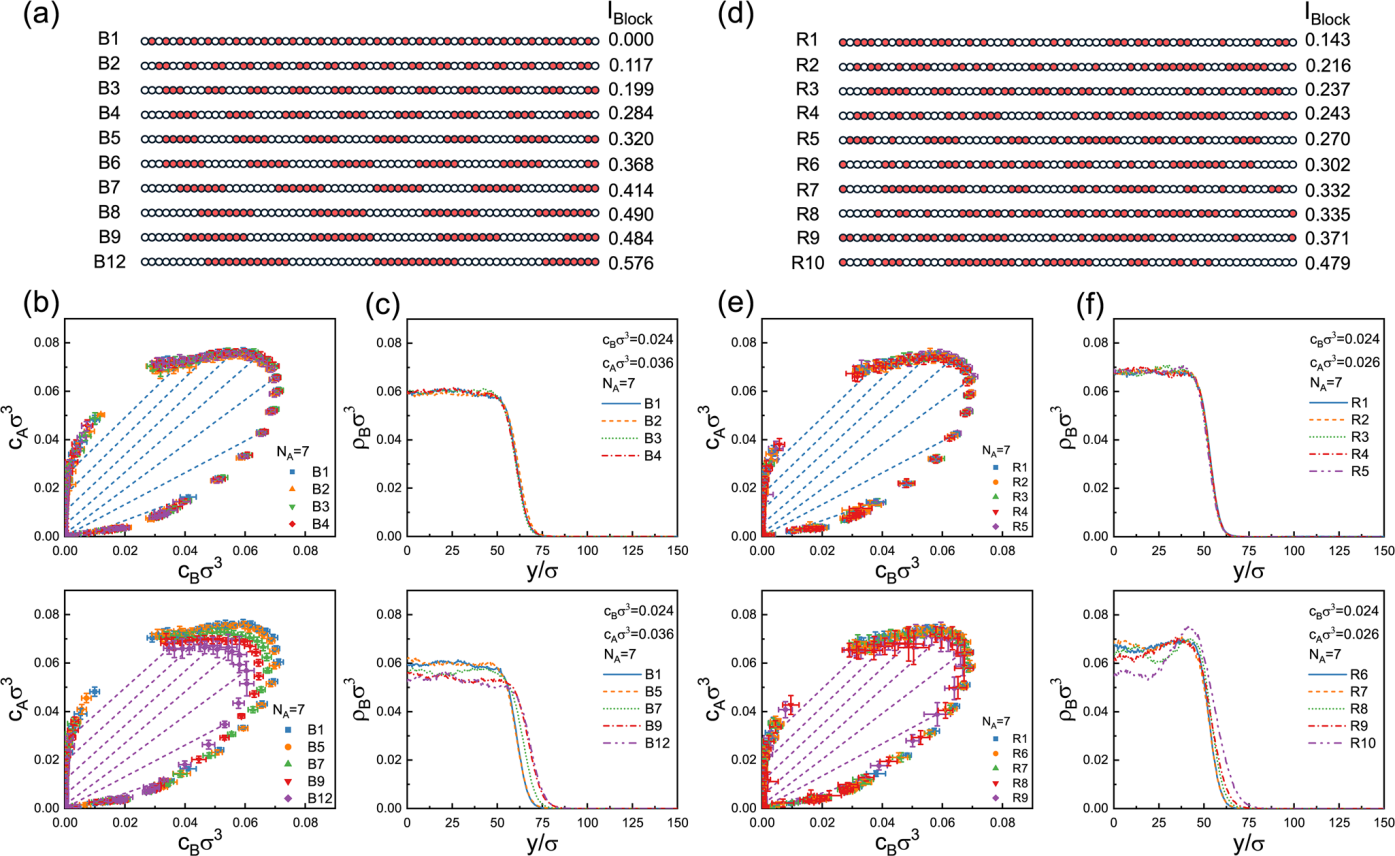
Phase diagrams and density profiles for systems with $N_{A}=7$ and varying sequence distribution of crosslinkable B‐monomers along the B‐chains: (a) Specification of regular sequences B1‐B12 with corresponding blocking number $I_{\text{Block}}$ (see text). (b) Phase diagrams for regular sequences B1‐B12 and (c) examples of corresponding density profiles from slab simulations. (d) Specification of irregular sequences R1‐R10 with corresponding blocking number $I_{\text{Block}}$. (e) Phase diagrams for irregular sequences R1‐R9 and (f) examples of corresponding density profiles.

Next we examined how the phase behavior depends on the distribution of crosslinkable monomers along the B‐chains. The A‐chain was fixed at $N_{A}=7$, while the B‐chains contained $f_{B}=32$ crosslinkable monomers arranged in either regular or irregular patterns (Figure 2a,d).

To quantify the blockiness of the sequences, we adopted the normalized “sequence charge decoration” (nSCD) concept, which was originally introduced to characterize charge patterns in intrinsically disordered proteins [42, 53, 54], and also recently applied to describe sticker patterns on copolymers with heteroassociative monomer interactions [45]. Following this idea, we defined a blockiness parameter $I_{\text{block}}$ characterizing a sequence $S=(\tau_{1},\text{…},\tau_{N_{B}})$, where $\tau_{i}=1$ if monomer i is crosslinkable and $\tau_{i}=0$ otherwise:

$$I_{\text{block}}=\frac{I\left[S\right]-I_{\min}}{I_{\max}-I_{\min}}\;\text{with}\;I\left[S\right]=\sum_{i<j}\frac{\tau_{i}\tau_{j}}{\left|i-j\right|}.$$

Here, $I_{\min}=48.94$ corresponds to the strictly alternating sequence shown in Figure 1, and $I_{\max}=97.87$ to a pure diblock sequence. The sequences used in this work with their $I_{\text{block}}$ parameter values are given in Figure 2, labeled as B1‐B12 (regular sequences, panel (a)) and R1‐R10 (irregular sequences, panel (d)), with blocking parameters ranging from zero to 0.5.

In protein condensate models where phase separation is driven by coacervation and solvent quality, sequence patterning has a pronounced effect on demixing phase diagrams. The phase separation propensity typically increases with increasing blockyness [24, 42, 45, 55]. By contrast, in our system, crosslink‐driven phase separation is remarkably insensitive to sequence variations up to $I_{\text{block}}∼0.4$. Notably, this implies that the predictions of the Semenov–Rubinstein theory [52] – which does not include sequence information – remain applicable across a surprisingly broad range of sequences.

Sequence effects do, however, manifest in the structure of interfaces between coexisting phases (see Figure S10 in Supporting Information), as surface segregation generally depends on the character of the end group [56, 57]. In our case, neutral domains are enriched at the interface, whereas the density profiles of crosslinkable monomers closely follow the overall density. Despite this segregation, we observe no measurable change in the interfacial width (see Supporting Information), suggesting that the interfacial tension also does not depend strongly on the sequence. Indeed, Pyo et al. [55] recently reported that interfacial tension in biocondensates is primarily governed by the system's proximity to a critical point.

To probe sequence effects further on a molecular level, we analyzed how they affect the number of A‐chains binding to B‐chains, as well as the frequencies of loops, bridges, and singly bound A‐chains (see Figure S6 in Supporting Information). These quantities were largely independent of the crosslink distribution, except for loop formation, which exhibited some variation but showed no clear correlation with $I_{\text{block}}$. Since phase separation is driven by bridges, these results demonstrate that the microscopic driving forces of crosslink‐driven condensation are not strongly affected by crosslinkable monomer sequence variations, provided the sequence is not too blocky.

### 2.3 Microstructure Formation

For highly blocky sequences with $I_{\text{block}}>0.4$, where block sizes are of the same order than the total chain length, the phase‐separated region in parameter space shrinks (Figure 2b,e, bottom). This is accompanied by enhanced density fluctuations within the dense phase, signaling the onset of microstructure formation within the dense phase. In the case of the most blocky irregular sequence R10, the density fluctuations were so pronounced (Figure 2f, bottom) that it was no longer possible to extract reliable phase boundaries from slab simulations.

**FIGURE 3:**
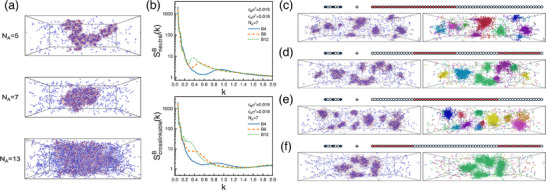
(a) Snapshots of systems with A‐chains of different lengths and B8‐chains (see Figure 2 for definition). (b) Structure factor of neutral (top) and crosslinkable (bottom) monomers on B‐chains in a system with $N_{A}=7$. (c–f) Snapshots of systems with very blocky B‐chains ($N_{A}=7$) exhibiting micellar structures: diblocks (c); tetrablocks (d); triblocks with neutral (e) and crosslinkable (f) outer blocks. Each snapshot is shown twice, once in regular representation with monomers colored by type (left), and once with chains colored by connected cluster. The volume is $100\times 300\times 80\sigma^{3}$.

Example snapshots from mixtures of B8‐chains ($I_{\text{block}}=0.49$) with A‐chains of different lengths are shown in Figure 3a, illustrating the emerging internal structure in the condensate. To quantify this effect, we calculated structure factors S(k) for neutral and crosslinkable B‐monomers using the Freud python package [58] in systems with regular B‐sequences of varying block lengths. The structure factor exhibits a clear peak at nonzero wavevector, which shifts toward smaller k as blockiness increases (Figure 3b). The corresponding radial distribution functions (RDFs) also reveal longer‐range correlations (see Figure S11 in Supporting Information) that extend beyond the initial oscillatory behavior caused by packing and excluded volume interactions. The emerging microdomain formation is also apparent from inspecting simulation snapshots as shown in Figure S12 (Supporting Information). The subdomains formed by crosslinkable monomers grow with increasing blockiness, and the monomer density in these subdomains is enhanced compared to the dense phase of less blocky polymers.

In the extreme case where B‐chains adopt a diblock copolymer architecture consisting of one crosslinkable monomer block and one neutral block, the condensates fragment into disconnected micelles. These micelles possess a core enriched in crosslinkable monomers and are stabilized by a corona of neutral chain blocks (Figure 3c). We further observed that similar disconnected micellar structures may also exist for B‐chains with tetrablock or triblock sequence architectures (Figure 3d,e). However, if large crosslinkable blocks are positioned at the two ends of B‐chains, the central part of the B‐chain can bridge between micelles, giving rise to a connected micellar network. This behavior is illustrated in Figure 3f (right), which shows two representative examples of systems with triblock B‐chains. In these snapshots, chains belonging to disconnected clusters are rendered in different colors. When the outer blocks of the B‐chains are neutral (Figure 3e), the system contains only disconnected micelles (each represented by a different color). By contrast, when the outer blocks are composed of crosslinkable monomers, all micelles are interconnected (uniform color), forming a network. As a result, the system undergoes phase separation into a dilute phase and a structured condensate composed of connected micelles.

Recently, Balzer and Fredrickson used the coherent‐state field theory (CSFT) framework [59] to study reversible crosslinking in homoassociative polymer solutions [60]. In doing so, they established CSFT as the field‐theoretic foundation of the Semenov–Rubinstein theory and showed how to naturally extend this mean‐field description to spatially inhomogeneous systems. Their analysis demonstrated that increasing sequence blockiness can induce microstructured gel phases, a trend that is qualitatively consistent with our observations.

## 3 Conclusion

In summary, our work establishes reversible crosslinking as a minimal yet powerful mechanism for liquid–liquid phase separation (LLPS), distinct from classical routes based on solvent‐quality changes or coacervation. Using coarse‐grained simulations supported by mean‐field Semenov–Rubinstein theory, we show that chain bridging via transient crosslinks alone can drive phase separation at ultralow polymer densities, yielding highly hydrated condensates stabilized by connectivity.

The phase diagrams reveal closed‐loop coexistence, re‐entrant phase separation, and sol–gel–sol transitions, all captured quantitatively by two parameters within the mean‐field framework, the dissociation constant and the effective repulsion parameter. Remarkably, the phase boundaries remain largely robust to sequence variations over a wide range of crosslink distributions, while extreme blockiness induces microphase separation into connected or disconnected micellar structures. Although the thermodynamic phase boundaries are largely insensitive to sequence architecture, we anticipate that dynamic properties will be far more affected [61].

We have employed a minimal coarse‐grained model – using an implicit solvent, neglecting long‐range electrostatics, and adopting a generic flexible chain description – to isolate the essential physics of entropy‐driven condensation in polymer systems with strong specific interactions. By establishing connectivity‐driven LLPS as a robust and tunable mechanism, this work provides a conceptual and quantitative foundation for understanding and exploiting crosslink‐mediated condensation in both natural and synthetic systems. When combined with additional weak interactions, the mechanism opens a rich design space for programmable soft materials, which will be interesting to explore in future work. For example, coupling additional weak interactions to reversible crosslinks, such as weak electrostatics, hydrophobic contacts, or π‐π/cation‐π motifs, can not only reshape the phase‐separation window [62, 63, 64], but also changes the lifetimes of reversible bonds. Controlling the latter while taking into account hydrodynamic interactions should allow manipulation of the viscoelastic properties and coarsening kinetics of the condensate [65], potentially stabilizing unconventional transient morphologies [66].

Furthermore, the ultralow density of these condensates opens practical opportunities. Their high water content makes condensates highly permeable to small molecules and ions and keeps macromolecular crowding low, leaving ample free volume for functionalization [67]. Relating solute partitioning and crosslinking reaction to network connectivity and bond lifetimes will translate these ideas into design rules for programmable soft materials – from membraneless separators to catalytic scaffolds – inspired by, yet not limited to, biological condensates.

## 4 Experimental Section

In our coarse‐grained simulations, we considered two types of polymer chains A and B in an implicit solvent. All monomers have the same mass m and diameter σ. Polymers A and B contain $f_{A}$ and $f_{B}$ crosslinkable monomers, respectively, which are connected by a harmonic bond potential $\beta U_{\text{bond}}=\frac{1}{2}k_{b}{\left(r-r_{0}\right)}^{2}$ and are subject to an additional bond angle potential $\beta U_{\text{bending}}=\frac{1}{2}k_{a}\;{\left(\theta -\pi \right)}^{2}$, where θ is the angle between consecutive bonds along a chain. Here $\beta =1/k_{B}T$ where $k_{B}$ is the Boltzmann constant, $k_{b}$ is the spring constant, $r_{0}=\sqrt[{6}]{2}\sigma$ is the equilibrium bond length in units of the monomer diameter σ, and $k_{a}$ is the bond angle parameter that tunes the rigidity of the polymers.

All monomers, except pairs of crosslinkable A‐ and B‐monomers, interact purely via a Weeks–Chandler–Andersen (WCA) potential [68]:

(1) $$\beta U_{\text{WCA}}=\left\{\begin{array}{ccc} & 4\varepsilon \left[{\left(\frac{\sigma }{r}\right)}^{12}-{\left(\frac{\sigma }{r}\right)}^{6}+\frac{1}{4}\right] & r<\sqrt[{6}]{2}\sigma \\ & 0 & r\geq \sqrt[{6}]{2}\sigma \end{array}\right.$$

where ε is the interaction strength. To mimic specific binding between crosslinkable A and B monomers, we introduce a short‐range attractive interaction of the form

(2) $$\beta U_{\text{binding}}=\left\{\begin{array}{ccc} & -\varepsilon_{\mathrm{sp}}\left[\cos\left(\frac{2\pi r}{\sigma }\right)+1\right] & r<0.5\sigma \\ & 0 & r\geq 0.5\sigma \end{array}\right..$$

This interaction ensures that a crosslinkable monomer A can bind to at most one crosslinkable monomer B. Once a bound pair has formed, further binding is prevented by repulsive WCA with the monomers of the pair [27, 45, 46, 69]. The strength of specific binding is controlled by the parameter $\varepsilon_{\mathrm{sp}}$.

We performed Langevin dynamics simulations at fixed temperature using the HOOMD‐blue simulation package (version 2.9.6) [70]. Snapshots shown in this work were visualized using OVITO [71]. The spring constant and angular potential constant were set to $k_{b}=30\;k_{B}T/\sigma^{2}$ and $k_{a}=5\;k_{B}T$, respectively, resulting in a persistence length of 5‐6 σ , and the strength of the WCA potential was set to $\varepsilon =1\;k_{B}T$. The binding strength was chosen to be $\varepsilon_{\mathrm{sp}}=6\;k_{B}T$, resulting in a binding lifetime $\tau ∼10^{3}\;t_{0}$, where the time unit is defined as $t_{0}=\sigma \sqrt{m/k_{B}T}$ (see Figure S2a in Supporting Information).

Systems were equilibrated for $0.5\times 10^{6}\;t_{0}$, which exceeds the decorrelation time of the end‐to‐end vector of the chains [72] in both the sol and gel states (see Figure S2c,d in Supporting Information). In a subsequent production run of length $0.5\times 10^{6}\;t_{0}$, 100 configurations separated by a time lag of $\Delta t=0.5\times 10^{4}\;t_{0}$ were extracted for data analysis. All results are based a 4–10 independent simulations, and error bars represent the standard error of the mean.

In order to calculate the phase diagram for different chain sequences, we employed slab simulations. The initial simulation box size was $30\times 30\times 30\sigma^{3}$. After $5\times 10^{4}\;t_{0}$, the box was extended along the y‐axis to 300σ, and chain positions were unwrapped along this direction when chains crossed periodic boundaries. The system was then equilibrated for an additional $0.5\times 10^{6}\;t_{0}$, followed by a production run of the same duration. Phase coexistence was identified both from the average density profiles along the y‐direction and from a block‐distribution analysis; both methods yielded identical coexistence lines. The phase diagrams reported here are based on density‐profile averaging.

### Statistical Analysis

All statistical analyses were performed on data obtained from at least four independent runs (100 frames per run) as explained above. Results are presented as mean ± standard deviation, with statistical differences analyzed using Origin 2021 (OriginLab, MA, USA). To determine the percolation transition boundaries, we calculated the percolation probability, P, defined as the probability of finding a system‐spanning cluster, for a wide range of concentrations of the two components. These data were visualized as heatmaps using Origin 2021 (OriginLab, MA, USA), where the color gradient represents the magnitude of averaged percolation probability ⟨P⟩ in the two‐parameter concentration space (e.g., $c_{A}$ vs. $c_{B}$). The percolation thresholds were then identified by extracting the contour lines corresponding to ⟨P⟩=0.5 from these heatmaps.

## Author Contributions

**Xinxiang Chen**: Conceptualization (equal); Methodology (equal); Software (equal); Formal Analysis (lead); Investigation (lead); Data Curation (lead); Writing – Original Draft (lead); Writing – Review & Editing (equal). **Jude Vishnu**: Software (equal); Writing – Original Draft (supporting). **Pol Besenius**: Conceptualization (supporting); Supervision (supporting); Writing – Review & Editing (supporting); Funding Acquisition (equal). **Julian König**: Conceptualization (supporting); Writing – Review & Editing (supporting); Supervision (supporting); Funding Acquisition (equal). **Friederike Schmid**: Conceptualization (equal); Methodology (equal); Formal Analysis (supporting); Resources (lead); Writing – Review & Editing (equal); Supervision (lead); Funding Acquisition (equal).

## Conflicts of Interest

The authors declare no conflicts of interest.

## Supporting information


**Supporting File**: advs74206‐sup‐0001‐SuppMat.pdf.

## Data Availability

The data that support the findings of this study are openly available in Zenodo at https://doi.org/10.5281/zenodo.17244719, reference number 17244719.
