# Supplementary material for: Dilute but Dense – Reversible Crosslinking Enables Water‐Rich (Bio)polymer Condensates
Source: Adv Sci (Weinh). 2026 Feb 4;13(20):e19636. doi: 10.1002/advs.202519636 (PMC13067872; doi:10.1002/advs.202519636)
Supplement: Supplementary file 1 — Supporting File: advs74206‐sup‐0001‐SuppMat.pdf. [file ADVS-13-e19636-s001.pdf]

Supporting Information: Dilute but dense – Reversible crosslinking enables water-rich (bio)polymer condensates

Xinxiang Chen,[*,†] Jude Ann Vishnu,[†,‡] Pol Besenius,[¶] Julian König,[§,‖] and Friederike Schmid[*,†]

†*Institute of Physics, Johannes Gutenberg-University Mainz, Mainz 55128, Germany*

‡*LPTMS, Université Paris-Saclay, France*

¶*Department of Chemistry, Johannes Gutenberg-University, 55099 Mainz, Germany*

§*Institute of Molecular Biology, 55128 Mainz, Germany*

‖*Theodor Boveri Institute, Biocenter, University of Würzburg, 97074 Würzburg, Germany*

E-mail: xichen@uni-mainz.de; friederike.schmid@uni-mainz.de

Contents

- Properties of the simulation model
- Theory Section
- Additional Figures
- References

Properties of the simulation model

The simulation model is described in the main paper, Experimental section. Here we add some additional information on properties of the model.

Influence of chain stiffness

The model used here is essentially identical to a model introduced in our earlier work, Ref. [S1], except that it includes the additional bond angle potential

$$\beta U_{\text{bending}} = \frac{1}{2} k_a (\theta - \pi)^2, \tag{S1}$$

In test simulations of a system of fully flexible chains (i.e., setting the bond angle parameter $k_a = 0$), we found that single B-chains may form artificial stable complexes with surrounding A-chains if crosslinkable A and B monomers are only separated by one or two neutral monomers. An example is shown in Figure S1(a). The key structural elements of such complexes are ultrashort loops between neighboring crosslinkable A- and B-monomers, which dominate the binding topology, such that A-chains no longer bridge between B-chains. As a result, phase separation is suppressed. This effect is an artifact of our bead–spring model and does not necessarily occur in real molecules, where side chains and steric constraints prevent extreme bending. Introducing a bending potential suppresses the complex formation and phase separation becomes possible, as shown in Figure S1(b). A structural analysis (Figure S1(c) and (e)) indicates that introducing chain stiffness does not change the total number of crosslinks but shifts the binding topology: Stiffness reduces loop formation and promotes bridge formation. Even a small bond angle potential ($k_a = 2\, k_B T$) is sufficient to enable phase separation, with coexistence densities (see Figure S1(d)) that are already very close to those obtained at larger stiffness ($k_a \geq 5\, k_B T$). Close to the rod-like limit ($k_a > 20\, k_B T$), the coexistence densities decrease again slightly.

To avoid the artificial complex formation in the case of short spacers, we introduce a small, but finite stiffness in our system ($k_a = 5\,k_BT$). The resulting persistence length of the chains is in the range of 5 beads, i.e., $5 - 6\sigma$. This is still much smaller than the contour length of the long B-chains, i.e., they are still flexible. We anticipate that the phase behavior might change in the rod-like limit, and the Semenov-Rubinstein theory might no longer be applicable. Studying this will be an interesting problem for future studies.

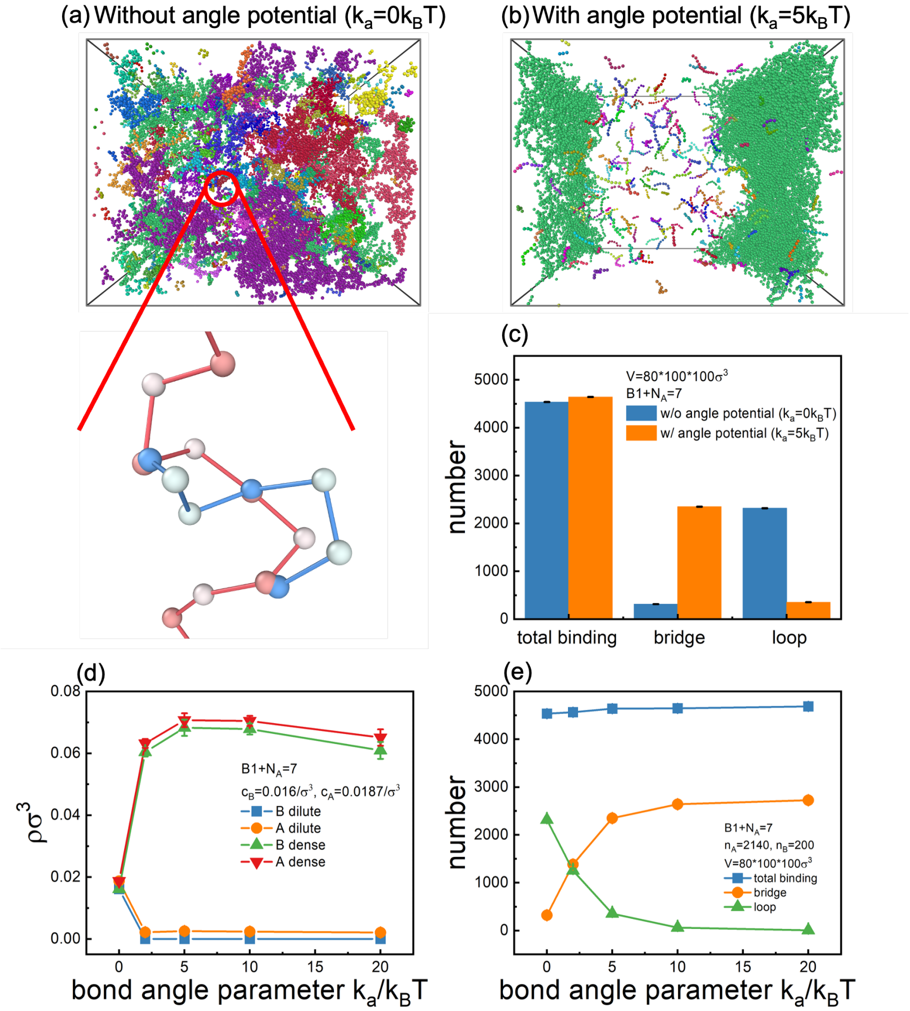

Figure S1: (a) and (b) Snapshots of a whole system of type B1 with $N_A = 7$ (see main text for classification) without and with bond angle potential at $c_B = 0.016/\sigma^3$ and $c_A = 0.0187/\sigma^3$. Disconnected clusters are coloured differently. (c) Number of total crosslinks, bridges, and loops in the same system. The error bar is obtained by averaging over 100 frames in the equilibrium state. (d) Coexistence densities of the two components as a function of the bond angle parameter k_a. (e) Number of total crosslinks, bridges, and loops as a function of k_a, averaged in equilibrium as in (c).

Time scales in the simulations

One important time scale in our system is the lifetime of a reversible bond. We determine it as a function of the binding strength ϵ_{sp} by studying a system containing one A chain and one B chain. The result is shown in Figure S2(a). At $\epsilon_{\text{sp}} = 6\,k_BT$, the binding lifetime is $\tau \sim 10^3 t_0$ in units of $t_0 = \sigma\sqrt{m/k_BT}$, which corresponds to the order of magnitude of lifetimes in real relatively dynamic systems.[S2]

In addition, we also calculated the autocorrelation function of end-to-end vector $\mathbf{R}(t)$, $P(t) = \langle \mathbf{R}(t)\mathbf{R}(0)\rangle / \langle \mathbf{R}(0)\mathbf{R}(0)\rangle$ for the pre-gel and post-gel states. The result is shown in Figure S2(c,d),[S3] demonstrating that our systems can fully relax in the simulation time. Therefore, in the following discussion, we keep $\epsilon_{\text{sp}} = 6\,k_BT$.

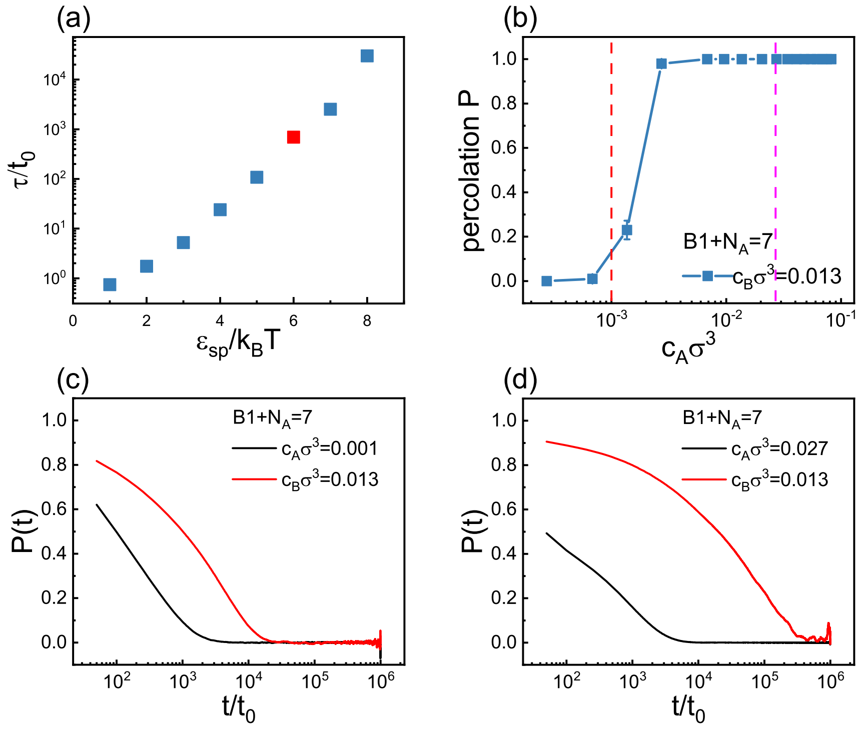

Figure S2: (a) Binding lifetime τ/t_0 as a function of binding strength $\epsilon_{\text{sp}}/k_{\text{B}}T$, with the red marker indicating the parameter $\epsilon_{\text{sp}} = 6k_{\text{B}}T$ used in our simulations. (b) Percolation probability P versus c_A; dashed lines denote the pre-gel and post-gel state points selected for dynamical analysis in (c,d). (c,d) Autocorrelation function of end-to-end vector $P(t)$ for the pre-gel and post-gel states, both showing full relaxation within the simulation window, confirming that the reversible network remains dynamical on simulation timescales.

Theory

Recapitulation of Semenov-Rubinstein theory

The Semenov-Rubinstein theory is a mean-field theory to study the relationship between phase separation and the sol-gel transition in associative polymer systems.[S4–S7] It has recently been extended to the two component case with specific binding interactions.[S8] Here we briefly recapitulate the Ansatz and the main equations. We consider a volume V containing two different polymer components in implicit good solvent, i.e., n_i i chains of length N_i containing f_i crosslinkable monomers, in which $i = A, B$. Crosslinkable monomers on chains of type A and B can reversibly bind to each other, however, every monomer can only form one such bond at a time. The binding energy between these two kinds of crosslinkable monomers is $-\epsilon_{\mathrm{sp}}$ (negative sign means it is an attractive interaction). Thus the free energy density of the whole system is composed of three parts: $\beta f = \beta f_{\mathrm{entropy}} + \beta f_{\mathrm{binding}} + \beta f_{\mathrm{int}}$. The first term is the pure translational entropy of polymer chains as a function of monomer concentration $c_i = n_i N_i / V$:

$$\beta f_{\mathrm{entropy}} = \frac{c_A}{N_A} \ln\left(\frac{c_A}{N_A e}\right) + \frac{c_B}{N_B} \ln\left(\frac{c_B}{N_B e}\right) \tag{S2}$$

The second term in the free energy density is due to the specific binding of crosslinkable monomers between A and B. Assuming there are N_P pairs forming in the reversible binding process, the free energy density $\beta f_{\mathrm{binding}}$ is calculated as follows:

$$\beta f_{\mathrm{binding}} = -\frac{1}{V} \ln\left[P_{\mathrm{comb}} \left(\frac{v_b}{V}\right)^{N_P} e^{\epsilon_{\mathrm{sp}} N_P}\right] \tag{S3}$$

Here v_b is the bond volume for specific binding, and P_{comb} is a combinatorial factor accounting for the number of different ways for the combination N_P pairs of binding between A and B, which is given by:

$$P_{\mathrm{comb}} = \binom{n_A f_A}{N_P} \binom{n_B f_B}{N_P} (N_P)! \tag{S4}$$

With Stirling's approximation, Eq. S3 can be approximated by

$$\begin{aligned}\beta f_{\text{binding}} &= c_P + \frac{f_A}{N_A} c_A \ln\left(1 - \frac{c_P}{c_A(f_A/N_A)}\right) + \frac{f_B}{N_B} c_B \ln\left(1 - \frac{c_P}{c_B(f_B/N_B)}\right) \\ &- c_P \ln \frac{(\frac{f_A}{N_A} c_A - c_P)(\frac{f_B}{N_B} c_B - c_P)}{c_P K}\end{aligned} \tag{S5}$$

with the binding pair concentration $c_P = N_P/V$ and the dissociation constant $K = v_b^{-1} e^{-\beta \epsilon_{\text{sp}}}$. The solvent-mediated part βf_{int} can be written in terms of the excluded volume interactions between monomers:

$$\beta f_{\text{int}} = \frac{1}{2} v_{\text{ex}} (c_A + c_B)^2 - v_{\text{ex}} \frac{f_A}{N_A} \frac{f_B}{N_B} c_A c_B \tag{S6}$$

where the parameter v_{ex} describes the effective repulsion between most monomers except crosslinkable A-B monomer pairs. Minimizing the free energy with respect to the binding number, $\partial f_{\text{binding}}/\partial c_P = 0$, we obtain the total free energy density

$$\begin{aligned}\beta f = &\frac{c_A}{N_A} \ln\left(\frac{c_A}{N_A e}\right) + \frac{c_B}{N_B} \ln\left(\frac{c_B}{N_B e}\right) \\ &+ c_P + \frac{f_A}{N_A} c_A \ln\left(1 - \frac{c_P}{c_A(f_A/N_A)}\right) \\ &+ \frac{f_B}{N_B} c_B \ln\left(1 - \frac{c_P}{c_B(f_B/N_B)}\right) \\ &+ \frac{1}{2} v_{\text{ex}} (c_A + c_B)^2 - v_{\text{ex}} \frac{f_A}{N_A} \frac{f_B}{N_B} c_A c_B\end{aligned} \tag{S7}$$

and the binding pair concentration at equilibrium c_P is determined from the implicit equation

$$\left(\frac{f_A}{N_A} c_A - c_P\right)\left(\frac{f_B}{N_B} c_B - c_P\right) = c_P K \tag{S8}$$

Based on the free energy function (Eq. S7), the phase behavior of this system can be analyzed in a standard method by equating the chemical potentials $\mu_i = \partial f/\partial(c_i/N_i)$ for each component and the osmotic pressure $\Pi = \sum_{i=A,B} \mu_i (c_i/N_i) - f = \sum_{i=A,B} c_i \partial f/\partial c_i - f$ in the two phases. Here, there are four variables with three equations. Since there is one degree of freedom, we need to fix one of the four variables to solve the equations. Here, we fix

the dense concentration of A component and solve the three equations numerically by using the Newton-Raphson method.[S9,S10] The coexistence curve is then constructed by scanning all possible values of the dense concentration of A component. The iterative process will be stopped when the residual error in $\max\{|\mu_i^{\text{dense}} - \mu_i^{\text{dilute}}|, |\Pi^{\text{dense}} - \Pi^{\text{dilute}}|\} < 10^{-12}$.

For the sol-gel transition, in the framework of Flory-Stockmayer theory,[S11,S12] assuming the network has a tree-like structure, the gelation condition for the two component system with heteroassociation is given by[S1,S8]

$$m = (f_A - 1)p_A \times (f_B - 1)p_B = m_p = 1. \tag{S9}$$

where $p_i = c_P/(f_i c_i/N_i)$ is the binding probability for each component.

Limit of vanishing dissociation constant, $K \to 0$

In the limit of zero dissociation constant, $K \to 0$, the binding pair concentration c_P as obtained from Eq. (S8) takes the simple form

$$c_P = \min\left(\frac{f_A}{N_A}c_A, \frac{f_B}{N_B}c_B\right) =: \min(c_{sA}, c_{sB}), \tag{S10}$$

where we have taken into account that c_P cannot exceed the concentrations of crosslinkable monomers $c_{sA} = \frac{f_A}{N_A}c_A$ and $c_{sB} = \frac{f_B}{N_B}c_B$ of type A and B, respectively. Further defining $c_\Delta = c_{sA} - c_{sB}$ and inserting the above result into Eq. (S5), we obtain the total free energy density $\beta f = \beta f_0 + c_P \ln(K)$ with

$$\begin{aligned} \beta f_0(c_P, c_\Delta) = &\frac{c_A}{N_A}\ln\left(\frac{c_A}{N_A e}\right) + \frac{c_B}{N_B}\ln\left(\frac{c_B}{N_B e}\right) \\ &- c_P \ln\left(\frac{c_P + |c_\Delta|}{e}\right) - |c_\Delta|\ln\left(\frac{c_P + |c_\Delta|}{|c_\Delta|}\right) \\ &+ \frac{1}{2}v_{\text{ex}}(c_A + c_B)^2 - v_{\text{ex}}\frac{f_A}{N_A}\frac{f_B}{N_B}c_A c_B \end{aligned} \tag{S11}$$

with $c_A = (c_P + \max(0, c_\Delta))N_A/f_A$ and $c_B = (c_P + \max(0, -c_\Delta))N_B/f_B$. At $K = 0$, the number N_P of AB pairs bound is fixed (maximal) for all concentrations. We can then omit the constant term $(v_b e^{\epsilon_{\mathrm{sp}}})^{N_P}$ in Eq. (S3) and repeat all calculations, giving $\beta f = \beta f_0$. Thus, the singular term $c_P \ln(K)$, which contributes a constant for each AB pair, can be safely omitted in the limit $K \to 0$.

Matching theory parameters to simulations

When comparing the theoretical predictions with the phase diagram obtained with simulations, we identify the dissociation constant K with

$$K = \left(\int \mathrm{d}\mathbf{r} \left[\exp(-\beta U_{\mathrm{binding}}) \right] \right)^{-1} \simeq 0.001257\sigma^{-3}, \tag{S12}$$

in which $\beta U_{\mathrm{binding}}$ is defined in Equation (1) in the main text.

To determine the effective repulsion parameter v_{ex}, we fit the phase boundary obtained from simulations of B1 chains with A chains of length $N_A = 7$. Each simulation coexistence point was projected onto the theoretical binodal using a nearest-distance criterion. For a given v_{ex}, the deviation between simulation and theory was quantified through a two-dimensional chi-square parameter,

$$\chi^2 = \sum_i \left[\frac{(c^A_{\mathrm{sim},i} - c^A_{\mathrm{th},i})^2}{\sigma^2_{A,\mathrm{sim},i}} + \frac{(c^B_{\mathrm{sim},i} - c^B_{\mathrm{th},i})^2}{\sigma^2_{B,\mathrm{sim},i}} \right], \tag{S13}$$

which incorporates statistical uncertainties and compares both coordinates of the binodal simultaneously. As shown in Figure S3(a), this procedure identifies the closest theoretical coexistence points for each simulation point. The minimum of χ^2 (Figure S3(b)) occurs at $v_{\mathrm{ex}} = 1.73\sigma^3$, which we therefore employ as the effective repulsion parameter in this work.

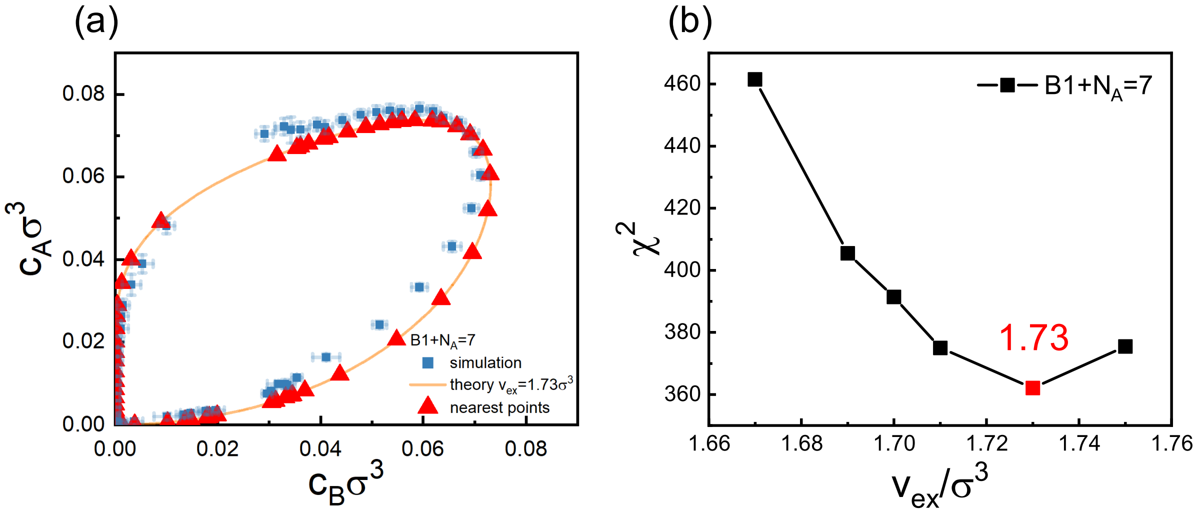

Figure S3: (a) Binodal line from simulation and theory with $v_{\text{ex}} = 1.73\sigma^3$. The red points represent the closest points from the theoretical line to the simulation points. (b) Two-dimensional chi-square, χ^2, as a function of the excluded-volume parameter v_{ex} for the B1 with $N_A = 7$ system. The minimum at $v_{\text{ex}} = 1.73\sigma^3$ indicates the best agreement between simulation and theory.

The theory assumes that the densities of crosslinkable monomers A and B are distributed homogeneously in space and neglects correlations due to chain connectivity, which seems like a very strong approximation. Nevertheless, Figure S4(a) demonstrates that the theory predicts the numbers of crosslinks (the binding numbers) at given monomer concentrations c_A and c_B quite accurately, even in the regime where the longer chains – B-chains – do not even overlap. Furthermore, it shows that for the parameters of the simulation, the binding numbers are close to the maximum binding number $N_p^{\max} = \min(n_A f_A, n_B f_B)$ (see Figure S4 (b)).

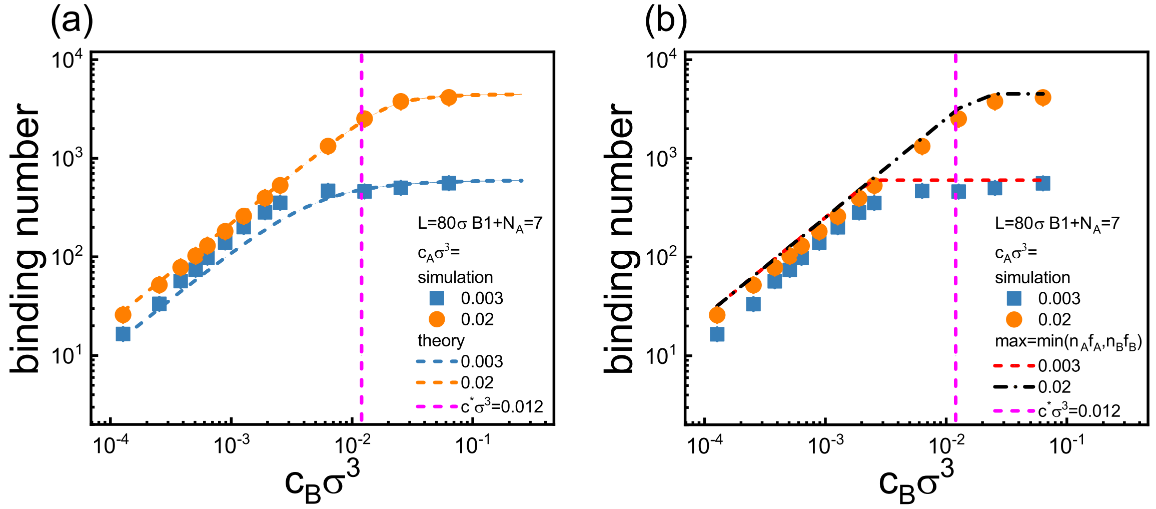

Figure S4: (a) Number of crosslinks (binding number) in the equilibrium system as a function of c_B for $c_A = 0.003/\sigma^3 < c^*$ (blue) and $c_A = 0.02/\sigma^3 > c^*$ (orange) for the B1 system with $N_A = 7$, compared with theory (dashed lines). The theoretical binding numbers are calculated from Eq. S8. (b) Same date compared with the corresponding maximum binding numbers at maximal saturation (black and red dashed curve for each case).

Additional Figures

This section provides additional figures which address technical aspects or provide further evidence for some of the statements in the main paper. They are referenced and discussed in the main paper.

Finite size effects on the bulk phase diagram

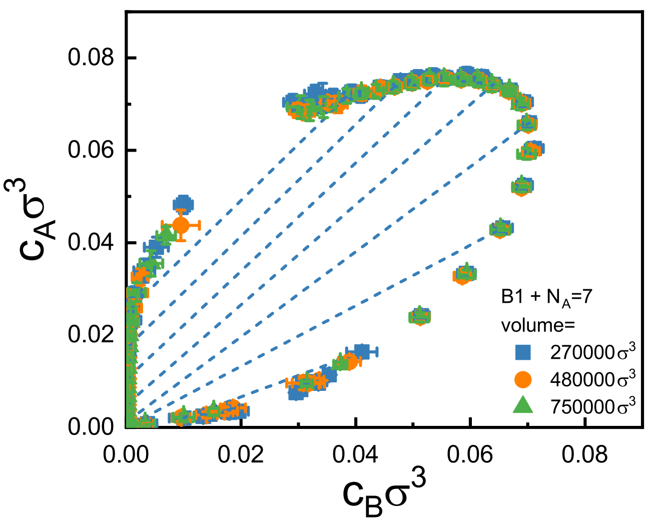

Figure S5: Coexistence curves for the system B1 with $N_A = 7$ system at different volumes to test finite size effects in our system. Only the case of $N_A = 7$ with B1 system is shown here, as the other cases are similar and are omitted for brevity.

Binding structures

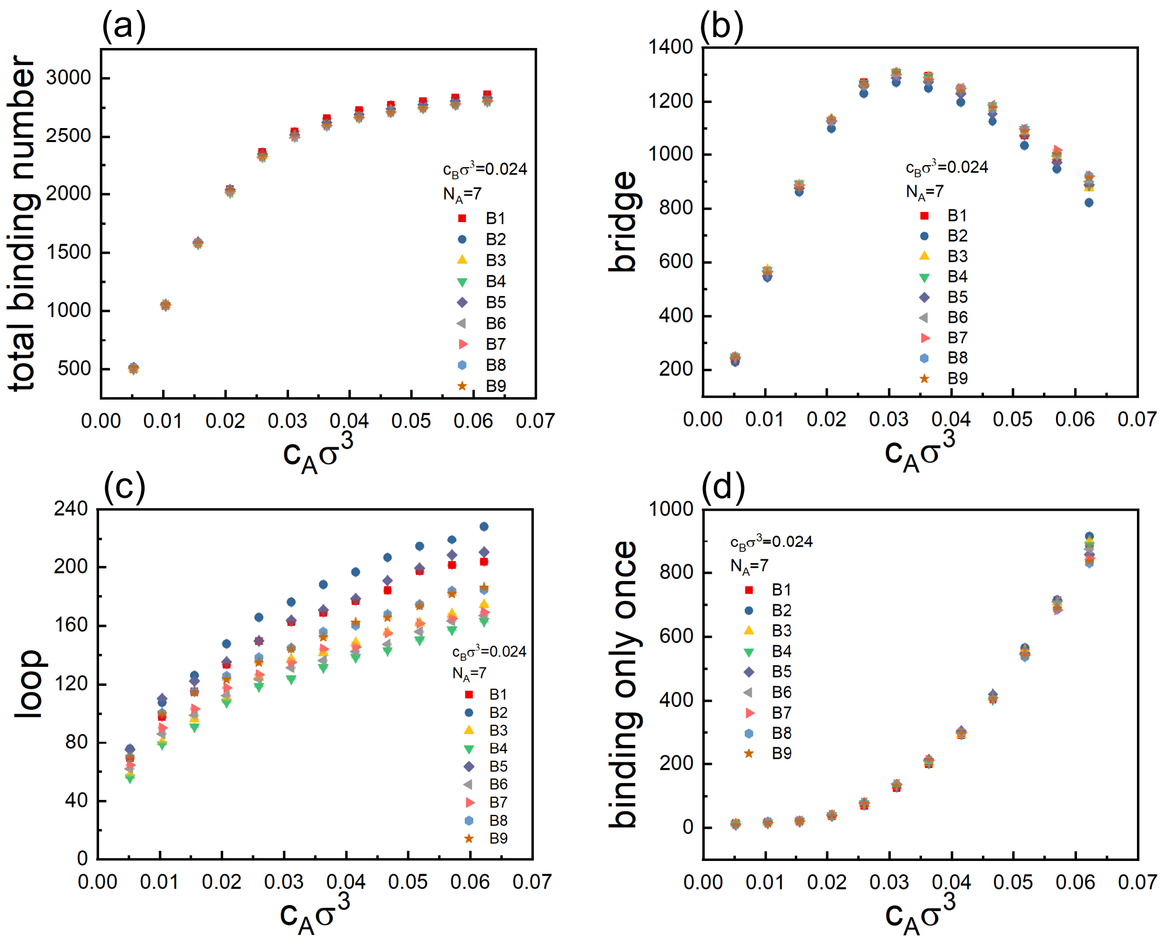

Figure S6: Total number of specific bonds and number of individual binding structures in the system containing $N_A = 7$ with different regular B sequences (see Figure 2(a), main text, for notation). Here $c_B = 0.024/\sigma^3$ is fixed. (a) Total number of bonds. (b) Bridges, where two crosslinkable monomers in one A chain bind to crosslinkable monomers from two different B chains respectively. (c) Loops, where two crosslinkable monomers on an A chain bind to two crosslinkable monomers on the same B chain. (d) "Binding only once", where an A chain binds only to one B chain.

Finite size effects on the percolation threshold

To calculate the percolation threshold, we follow the standard definition of percolation and identify a spanning cluster by requiring that a closed path within the cluster can be traced from any particle to its periodic image. We set $P = 1$ if at least one spanning cluster exists in the system and $P = 0$ otherwise; the percolation threshold is then determined from the condition $P = 0.5$. Figure S7 shows the effect of the simulation box size and shape when following this procedure.

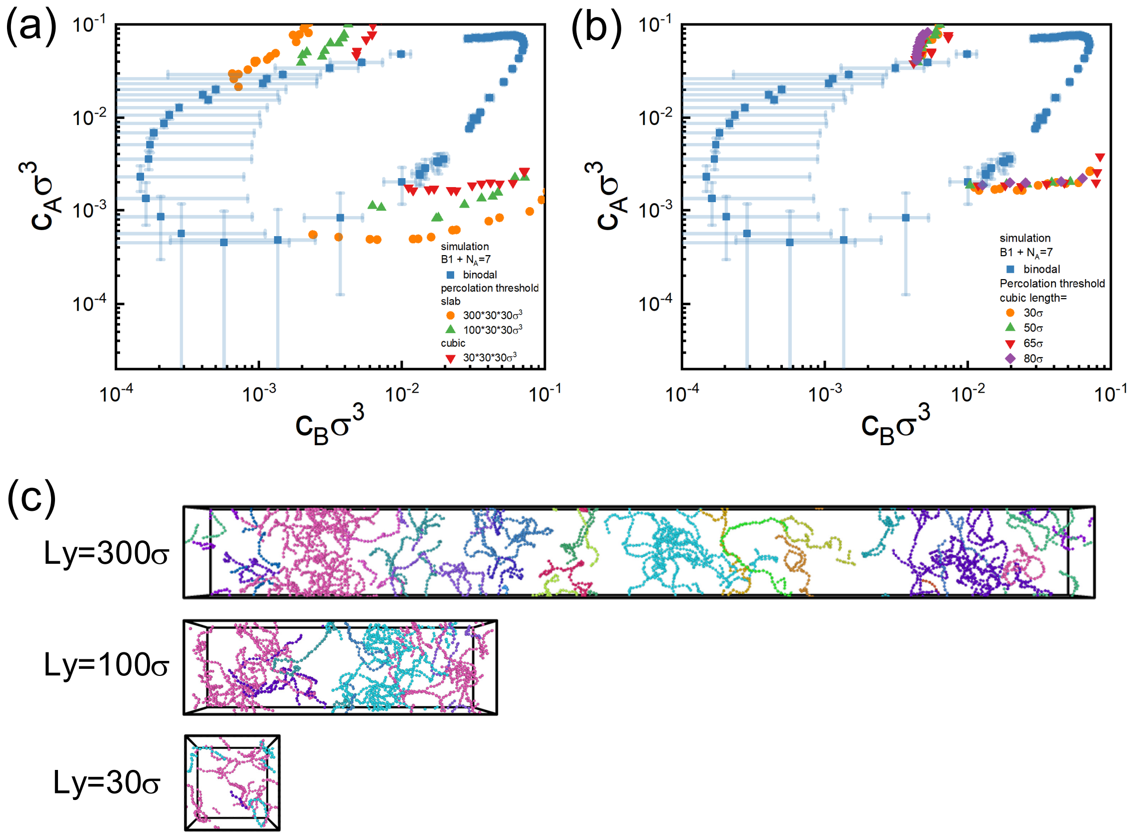

Figure S7: Calculated percolation threshold in systems with different volumes to test finite size effects in the $N_A = 7$ with B1 system. Blue points shown the coexistence curve for $N_A = 7$ with B1 case (a) Results from slab simulation, (b) Results from simulations in cubic boxes with different volumes. (c) Snapshots from equilibrated slab simulations at the concentration $c_B = 0.01/\sigma^3$ and $c_A = 0.001/\sigma^3$, in which the spanning cluster is colored pink.

Size of second largest cluster at the percolation threshold

One signature of the percolation threshold which is also sometimes used to identify percolation is a pronounced drop of the size of the second largest cluster.[S13] We have tested this effect in our model. To this end we calculate a quantity $\phi_{\text{cluster}}^{\text{submax}}$, defined as the number of chains in the second-largest cluster, normalized by the total number of chains in the system. The results are shown in Figure S8. The purple line indicates the percolation threshold as obtained from calculating the order parameter P of percolation. The symbols correspond to individual simulations and are colored according to the value of $\phi_{\text{cluster}}^{\text{submax}}$. The percolation threshold (purple line) lies close to the peak of the second-largest cluster size. We further tested a system that exhibits only a percolation transition, shown in Figure S8 (b). In this case, percolation coincides with a sharp decrease in the size of the second-largest cluster.

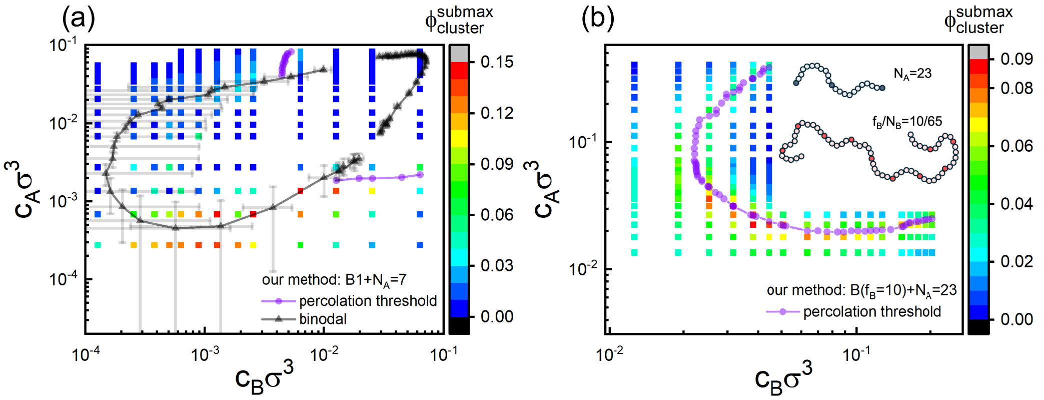

Figure S8: (a) The fraction of second-largest cluster for B1 and $N_A = 7$ system in which phase separation and percolation all happen. The back point-line is the binodal line, and the purple one is percolation threshold. (b) The fraction of second-largest cluster for B with $f_B = 10, N_B = 65$ and $f_A = 3, N_A = 23$ system, whose chain structure for chains is shown in the right corner. In this system, only percolation transition happens for different concentrations.

Comparison between simulation results and theoretical predictions

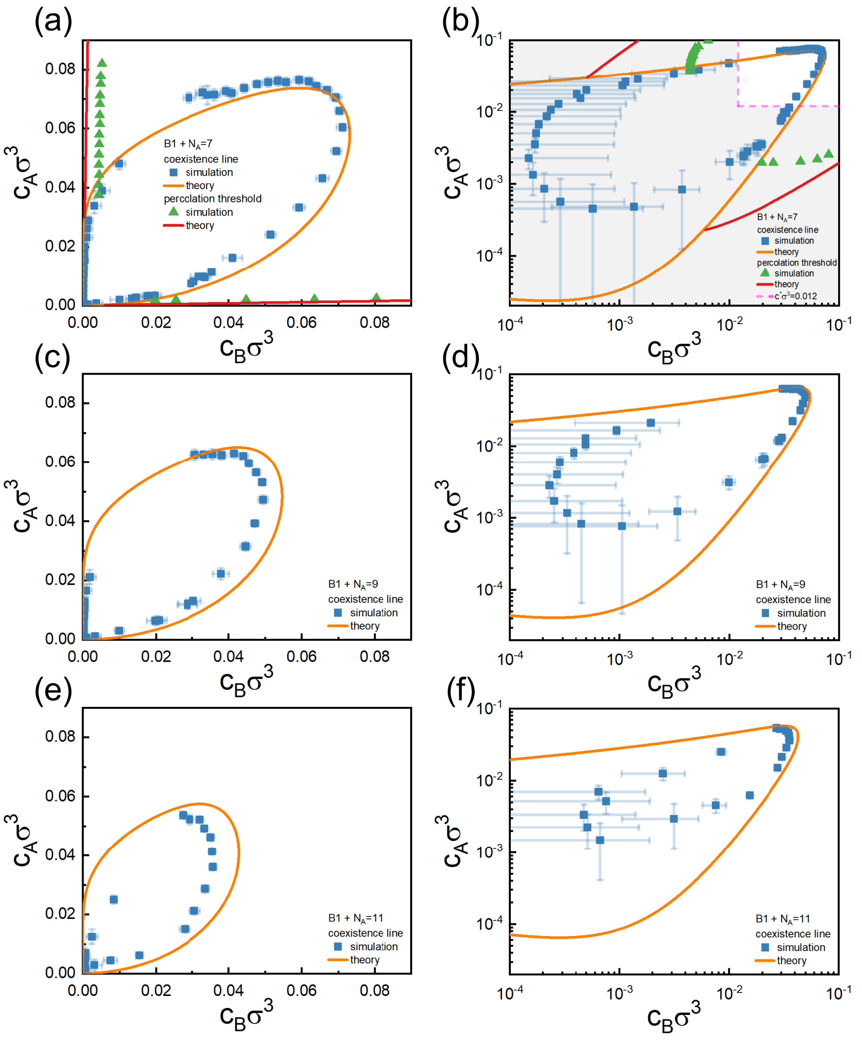

Figure S9: Comparison of the coexistence curve and the percolation threshold as obtained from simulations (symbols) and theory (lines) in the B1-system with A-chains of length $N_A = 7$ (a,b), $N_A = 9$ (c,d), $N_A = 11$ (e,f) in different representations. The left column shows the linear representation, the right column the double logarithmic representation. The theoretical predictions use the effective repulsion parameter $v_{\text{ex}} = 1.73\sigma^3$. The pink dashed line in (b) shows the boundary $c_A, c_B = c^*$ for the case of B1 with $N_A = 7$ (see main text). The grey shading marks the region where the mean-field criterion $c_{A,B} > c^*$ is not met.

Interfacial properties

While the thermodynamic phase envelopes are robust to the placement of crosslinkable monomers, the interfacial and dynamic properties are expected to be sensitive to the specific architecture. Unfortunately, calculating the interfacial tension with standard methods (pressure anisotropy) is not feasible for our ultra-dilute slabs, because it requires pressure data with very high statistical accuracy. Therefore, in order to assess the possible impact of sequence variations on the interfacial structure and interfacial tension, we have calculated two quantities that can provide indirect insights: (i) the density profiles of chain ends for different sequences, and (ii) the interfacial width, which is expected to be anticorrelated with the interfacial tension. The results are shown in Figure S10.

As shown, e.g., by Hill et al.[S14] and Lee et al.,[S15] even subtle changes in end-group functionalization can drive significant surface depletion or segregation, thereby altering interfacial properties. To assess this effect, we compare the end-segment distributions for two examples in Figure S10 (a,b): Systems of B-chains with B1 (a) and B3 (b) sequences mixed with A-chains of length $N_A = 7$ (for notation see Figure 2(a) in the main text). In the sequence B1 (Figure S10 (a)), both ends are spacer monomers, whereas in the sequence B2 (Figure S10 (b)), one end is a spacer monomer and the other a crosslinkable monomer. Figure S10 illustrates that spacer ends tend to enrich at the interface, whereas the density profile of crosslinkable end monomers follows the overall density profiles.

To estimate the impact of this on the interfacial tension, we analyze the interfacial width. Figure S10 (c) shows the apparent interfacial width (including capillary wave effects) as a function of blockiness for all regular sequences, which is calculated by fitting the total density profile along the y-axis to the expression

$$\rho(y) = \frac{1}{2}(\rho_{\text{dense}} + \rho_{\text{dilute}}) + \frac{1}{2}(\rho_{\text{dense}} - \rho_{\text{dilute}}) \tanh\left(\frac{2y}{L}\right).$$

The interfacial width is roughly constant within the error except for the two most blocky

sequences B9 and B12. When looking at the parameter $\gamma = (\rho_{\text{dense}} - \rho_{\text{dilute}})^2/L$, which would be proportional to the surface tension in a simple Cahn-Hilliard theory, we find that it starts decreasing for sequences with blockiness beyond B5, which is where the phase coexistence region starts shrinking. Thus our results suggest that blockiness only affects the interfacial tension in the (highly blocky) regime where it also affects the phase boundary. This is compatible with reports by Pyo et al.[S16] in a different system (homoassociative condensates without explicit spacers), who found that the interfacial tension is essentially determined by the proximity of the system to a critical point – although in their system, the phase separated region expands with increasing blockiness.

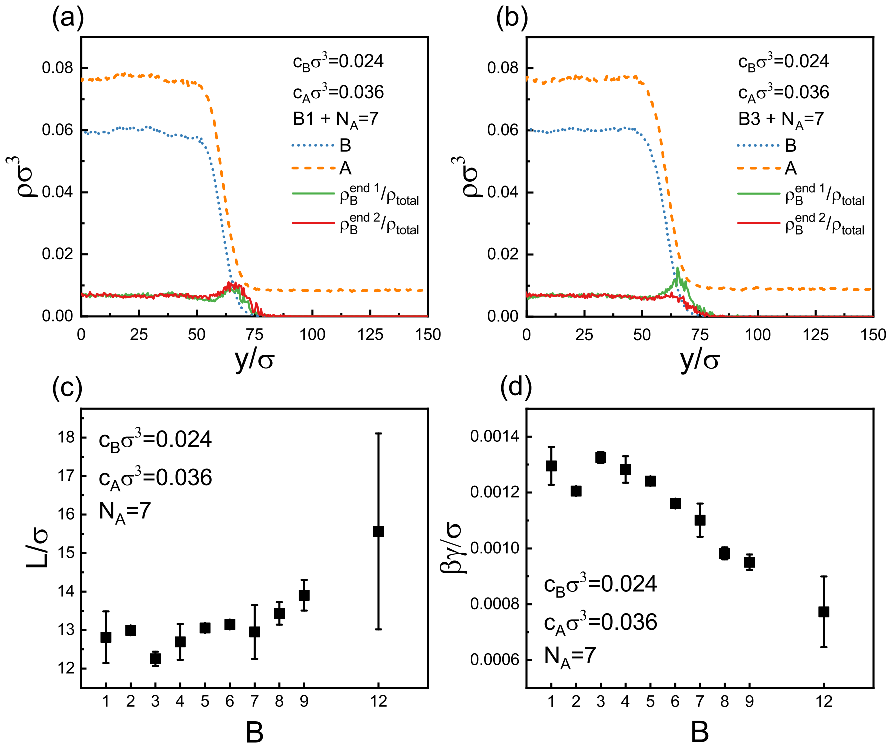

Figure S10: Monomer density $\rho(y)$ for systems of B1-chains (a) and B3-chains (b) with A chains of length $N_A = 7$ at $c_B = 0.024/\sigma^3$ and $c_A = 0.036/\sigma^3$. The green and red lines are the corresponding the reduced distributions for both end segments. (c) and (d) Interfacial width L and surface parameter $\gamma = \Delta\rho^2/L$ – $\Delta\rho$ is the density difference between the two phases – for regular B sequences with A-chains of length $N_A = 7$. In a simple Cahn-Hilliard type theory of phase separating fluids, the surface parameter would be proportional to the interfacial tension.

Radial distribution functions

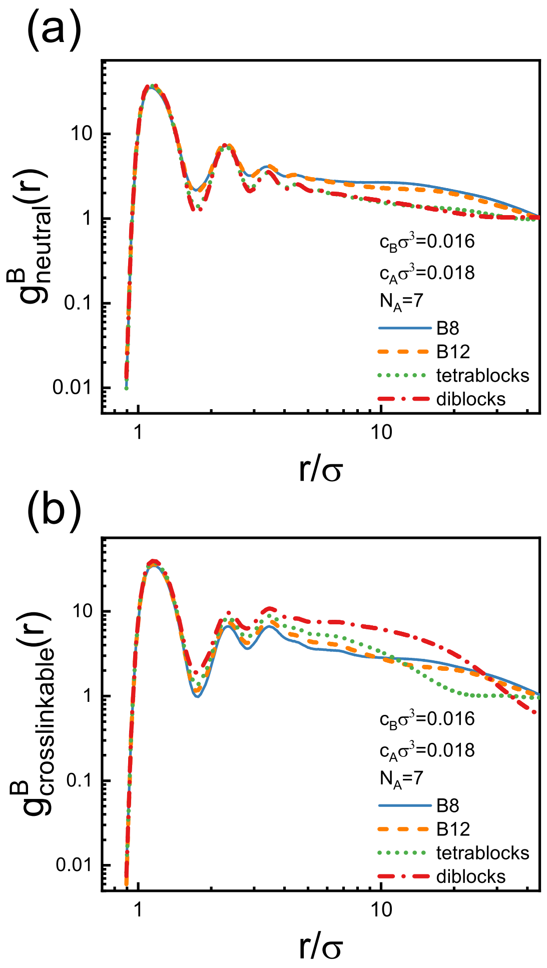

Figure S11: Radial distribution function of neutral-neutral (a) and crosslinkable-crosslinkable (b) monomers on B-chains for different B blocking cases. The overall concentration is $c_A = 0.018/\sigma^3$ for A monomers, and $c_B = 0.016/\sigma^3$ for B monomers.

Microphase separation

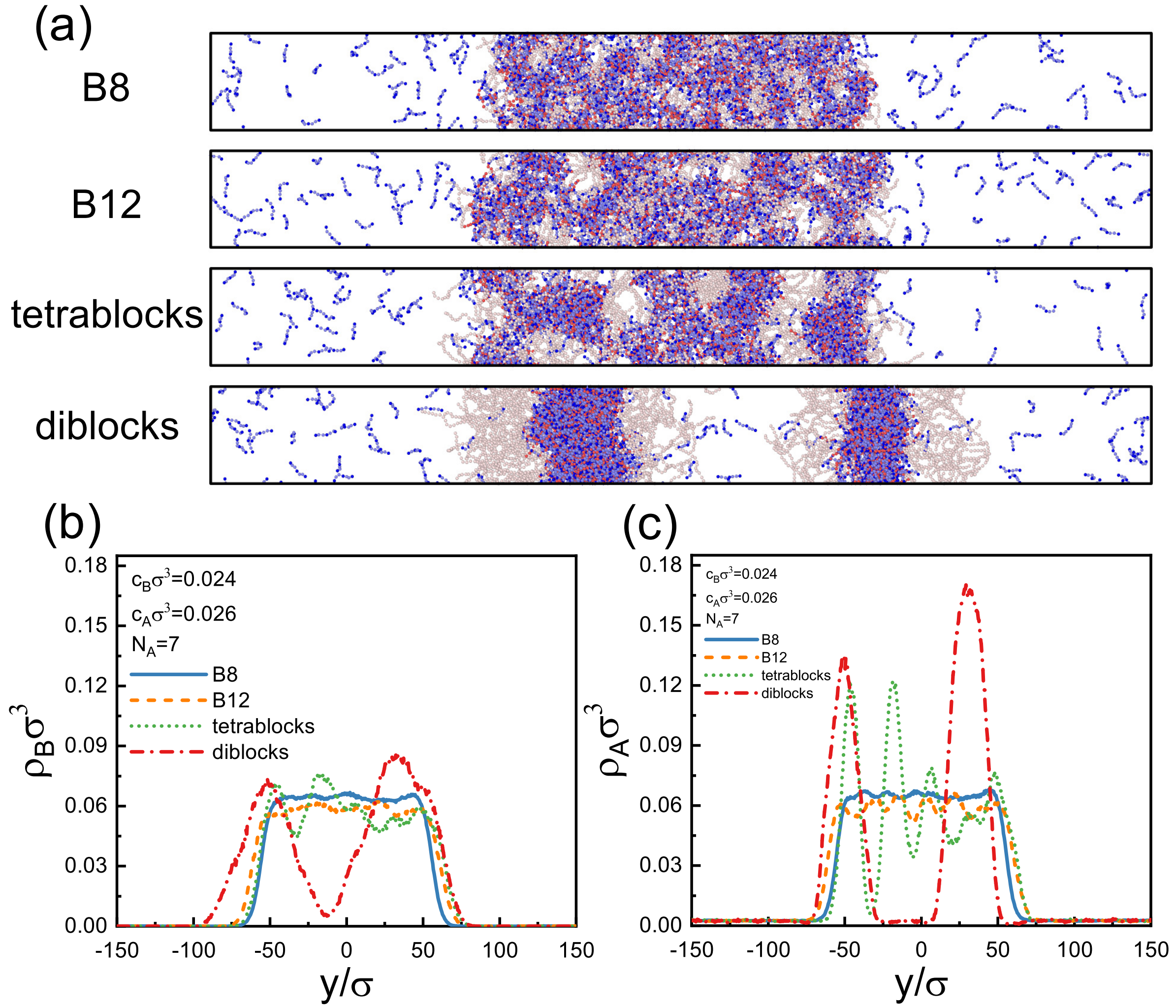

Figure S12: (a) Simulation snapshots of systems with $N_A = 7$ and B-chains of type B8, B12, diblocks, tetrablocks in the equilibrium state. The structure details of diblocks and tetrablocks are shown in Figure 3(c) and (d). The overall concentration for A is $c_A = 0.026/\sigma^3$ and for B, $c_B = 0.024/\sigma^3$. (b) and (c) The corresponding monomer density profile for B and A components, respectively.

References

(S1) Chen, X.; Vishnu, J. A.; Besenius, P.; König, J.; Schmid, F. Sol–Gel Transition in Heteroassociative RNA-Protein Solutions: A Quantitative Comparison of Coarse-Grained Simulations and the Semenov–Rubinstein Theory. *Macromolecules* **2025**, *58*, 3331–3342.

(S2) Galvanetto, N.; Ivanović, M. T.; Chowdhury, A.; Sottini, A.; Nüesch, M. F.; Nettels, D.; Best, R. B.; Schuler, B. Extreme dynamics in a biomolecular condensate. *Nature* **2023**, *619*, 876–883.

(S3) Behbahani, A. F.; Schmid, F. Relaxation Dynamics of Entangled Linear Polymer Melts via Molecular Dynamics Simulations. *Macromolecules* **2024**, *58*, 767–786.

(S4) Semenov, A. N.; Rubinstein, M. Thermoreversible gelation in solutions of associative polymers. 1. Statics. *Macromolecules* **1998**, *31*, 1373–1385.

(S5) Prusty, D.; Pryamitsyn, V.; Olvera de la Cruz, M. Thermodynamics of associative polymer blends. *Macromolecules* **2018**, *51*, 5918–5932.

(S6) Choi, J.-M.; Hyman, A. A.; Pappu, R. V. Generalized models for bond percolation transitions of associative polymers. *Phys. Rev. E* **2020**, *102*, 042403.

(S7) Michels, J. J.; Brzezinski, M.; Scheidt, T.; Lemke, E. A.; Parekh, S. H. Role of solvent compatibility in the phase behavior of binary solutions of weakly associating multivalent polymers. *Biomacromolecules* **2021**, *23*, 349–364.

(S8) Danielsen, S. P.; Semenov, A. N.; Rubinstein, M. Phase Separation and Gelation in Solutions and Blends of Heteroassociative Polymers. *Macromolecules* **2023**, *56*, 5661–5677.

(S9) Press, W. H. *Numerical recipes 3rd edition: The art of scientific computing*; Cambridge university press, 2007.

(S10) Zhang, P.; Alsaifi, N. M.; Wu, J.; Wang, Z.-G. Polyelectrolyte complex coacervation: Effects of concentration asymmetry. *J. Chem. Phys.* **2018**, *149*.

(S11) Flory, P. J. *Principles of polymer chemistry*; Cornell university press, 1953.

(S12) Stockmayer, W. H. Theory of molecular size distribution and gel formation in branched-chain polymers. *J. Chem. Phys.* **1943**, *11*, 45–55.

(S13) Jedlinska, Z. M.; Riggleman, R. A. Effects of Associative Interactions on the Phase Behavior of Complex Coacervates. *Macromolecules* **2024**, *57*, 4323–4334.

(S14) Hill, J. A.; Endres, K. J.; Meyerhofer, J.; He, Q.; Wesdemiotis, C.; Foster, M. D. Subtle end group functionalization of polymer chains drives surface depletion of entire polymer chains. *ACS Macro Lett.* **2018**, *7*, 795–800.

(S15) Lee, H.; Lee, Y.; Kim, N.; Park, M. J. Polymer chain-end chemistry: Unlocking next-generation functional materials. *Prog. Polym. Sci.* **2025**, 102003.

(S16) Pyo, A. G.; Zhang, Y.; Wingreen, N. S. Proximity to criticality predicts surface properties of biomolecular condensates. *Proc. Natl. Acad. Sci. U.S.A.* **2023**, *120*, e2220014120.
